# Potential public health hazards related to consumption of poultry contaminated with antibiotic resistant *Listeria monocytogenes* in Egypt

**DOI:** 10.1186/s12866-024-03183-x

**Published:** 2024-01-29

**Authors:** Amira Ibrahim Zakaria, Rana Fahmi Sabala

**Affiliations:** Department of Food Hygiene, Safety and Technology, Faculty of Veterinary Medicine, Mansoura University, Mansoura, 35516 Egypt

**Keywords:** Antibiotic resistance, Foodborne pathogens, *Listeria monocytogenes*, Public health hazards, Virulence genes

## Abstract

*Listeria monocytogenes* is an important foodborne pathogen that incorporated into many serious infections in human especially immunocompromised individuals, pregnant women, the elderly, and newborns. The consumption of food contaminated with such bacteria is considered a source of potential risk for consumers. Therefore, a total of 250 poultry purchased in highly popular poultry stores besides 50 swabs from workers hands in the same stores, in Mansoura City had been tested for the *L. monocytogenes* prevalence, virulence genes, and antibiotic resistance profile illustrating the health hazards from such poultry. The *L. monocytogenes* were recovered from 9.6% of poultry samples while not detected from workers hand swabs. The antimicrobial susceptibility of 24 *L. monocytogenes* strains against 24 antibiotics of seven different classes revealed high susceptibility rates to erythromycin (79.17%), streptomycin (66.67%), gentamycin (66.67%), vancomycin (58.33%), chloramphenicol (58.33%) and cefotaxime (41.67%). The majority (79.2%) of *L. monocytogenes* were classified as multidrug resistant strains with high resistance to tetracyclines and β-lactams antibiotics while 16.7% of the strains were categorized as extensively resistant ones. The *iap* virulence-specific determination gene had been detected in all recovered *L. monocytogenes* isolates while 83.33 and 70.83% of the isolates harbored *hylA* and actA genes. In addition, the study confirmed the capability of most *L. monocytogenes* isolates for biofilm formation by moderate to strong production and the quantitative risk assessment illustrated the risk of developing listeriosis as the risk value exceeded 100. The current results illustrate that poultry meat can be a source of pathogenic antibiotic resistant strains that may cause infection with limited or no treatment in immunosuppressed consumers via the food chain.

## Introduction

*Listeria monocytogenes* is one of the foodborne pathogens incorporated in many outbreaks worldwide [1–4]. *Listeria monocytogenes* causes human listeriosis usually of mild illness treated with antibiotics. Serious form of listeriosis primarily affects people who are at greater risk such as pregnant women resulting in severe disease in the fetus or even stillborn, people aged 65 years old or older, and people whose immune systems are very weak [5]. Invasive human listeriosis includes symptoms including septicemia, abortion, meningitis, meningoencephalitis, and even death as recorded to be one in five people with the infection [6]. The mortality rates associated with human listeriosis from food sources had been taken great concern worldwide as theyreached around 20 to 30% [7, 8].

*Listeria monocytogenes is* a Gram-positive, facultative anaerobic bacteria widely distributed in the environment [9, 10]. Although they are distributed in many sources, listeriosis usually arises after the consumption of contaminated food, such as undercooked food, ready-to-eat meals, and dairy products because of the capability of *L. monocytogenes* to grow at refrigeration temperatures (4 °C) and tolerate salty or acidic conditions [11]. *L. monocytogenes* has been frequently isolated from various food of animal origin, with high prevalence rates. In the poultry chain, contamination of poultry meat often occurs during slaughtering and processing, leading to its association with listeriosis outbreaks. Notably, a severe outbreak in South Africa in 2018 resulted in numerous cases and deaths due to the consumption of contaminated processed meat [12].

The pathogenicity of *L. monocytogenes* is promoted through various virulence factors [13] which mainly depend on its ability to invade and replicate within host cells, which attack the host immune system and spread throughout the body, especially in people of high risk. Virulence factors produced by *L. monocytogenes* involve *hlyA*, *actA,* and *iap* genes. The *hylA* is responsible for the invasion of host cells and the escape from the phagosomes [14, 15]. The actin assembly gene *actA* contributes tocell-to-cell spread [15]. The invasion-associated protein gene *iap* is involved in the adhesion and invasion of pathogens to the host cells [16]. The biofilm formation capability of listerial cells is also considered a key survival strategy for *L. monocytogenes*, contributing to its persistence in various environments and associated with its virulence to cause infection resistance to antimicrobial treatments [17].

In Egypt, studies have reported the presence of *L. monocytogenes* in various food products, including minced meat, fish fillets, sausage, and raw milk [10, 18, 19]. The consumption of such contaminated food is considered a potential risk for consumers. Therefore, the objectives of this study are to investigate the prevalence of *L. monocytogenes* in both poultry meat and workers’ hands and characterize their virulence genes, antibiotic resistance profile, and biofilm formation capability. Furthermore, the study highlights the health risks associated with consuming poultry products contaminated with *L. monocytogenes* which is crucial for food safety and public health.

## Material and methods

### Sample collection

Two hundred and fifty whole chicken carcass samples were collected from retail poultry shops located in Mansoura City, Egypt. On the other side, hand swabs were collected from fifty workers present in the same shops. Each sample of the chicken carcass was wrapped individually in a polyethylene bag, while the hand swabs were transferred in 25 ml of buffer peptone water, each and all the samples were transferred rapidly in an icebox to Meat Hygiene Laboratory, Faculty of Veterinary Medicine, Mansoura University located in Mansoura City. All the samples were analyzed bacteriologically for the presence of *L. monocytogenes*.

### Isolation and identification of *L. monocytogenes*

Detection of *L. monocytogenes* was performed as described by ISO 11290-1 for the isolation of such bacteria from food [20]. First for the enrichment of *L. monocytogenes*, 25 g of each poultry sample was diluted in 225 ml of Half Fraser broth (Oxoid, UK) and homogenized in a blender for 2 minutes. The hand swab samples were transferred to 10 ml of Half Fraser broth. Homogenates of poultry samples and swabs were incubated at 30 °C for 24 h. After that, 0.1 ml of pre-enriched culture was added to 10 ml of Fraser broth and incubated at 30 °C for 24 h. Each Fraser broth culture was streaked onto Palcam agar (Oxoid) and incubated at 37 °C for 48 h. Approximately five colonies of the growing *Listeria* specieswere purified and underwent further biochemical identification using catalase test, oxidase test, sugar fermentation test, and evaluation of hemolysis type [21]. The biochemically confirmed strains of *L. monocytogenes* in the present study were further verified using the API Listeria test (BioMerieux).

### Molecular analysis

Extraction of genomic DNA from the obtained isolates was performed according to Alexopoulou et al., [22]. In brief, overnight bacterial cultures were boiled for 15 min and centrifuged for 3 min at 10000 g. The supernatant was used as a DNA template and stored at − 20 °C. Molecular identification of *L. monocytogenes* was done by screening the 16S rRNA gene of 938 bp (Table 1) [23]. The PCR was set for 20 μl reaction volume using 0.1 μl of each primer (100 μmol) using Quick Taq™ polymerase by 10 μl with DNA template 1 μl. The amplification of the 16SrRNA was performed using an initial denaturation step at 94 °C for 2 min, followed by 25 cycles (94 °C for 30s denaturation, 57 °C for 30s annealing, and 68 °C for 1 min. extension). The final extension was performed at 68 °C for 10 min and held at 4 °C. The multiplex PCR reaction targeting the virulence genes (*hylA*, *actA*, and *iap*) was performed for positive isolates for the 16S rRNA gene by the same previous method except using an annealing temperature of 60 °C [24]. PCR amplification products were run on a 1.5% agarose gel by electrophoresis and photo-documented under an ultraviolet illuminator. PCR primer sequences used for the detection of *L. monocytogenes* virulence genes are illustrated in Table 1. *L. monocytogenes* ATCC 35152 strain was used as a positive control.

**Table 1:** PCR primer sequences used for the molecular identification of *L. monocytogenes* isolates and detection of their virulence genes

Target gene	Primer name	Oligonucleotide sequences	Amplicon (bp)	Reference
***16S rRNA***	*16S rRNA* (F) *16S rRNA* (R)	5′- CAGCAGCCGCGGTAATWC-3′ 5′- CTCCATAAAGGTGACCCT − 3’	938	[23]
***iap***	*iap* (F)	5′ ACAAGCTGCACCTGTTGCAG ′3	131	[24]
	*iap* (R)	5′ TGACAGCGTGTGTAGTAGCA ′3		
***hylA***	*hlyA* (F)	5′ GCAGTTGCAAGCGCTTGGAGTGAA ′3	456	[24]
	*hlyA* (R)	5′ GCAACGTATCCTCCAGAGTGATCG ′3		
***actA***	*actA* (F)	5′ CGCCGCGGAAATTAAAAAAAGA ′3	839	[24]
	*actA* (R)	5′ ACGAAGGAACCGGGCTGCTAG ′3		

### Antibiotic susceptibility testing

The antimicrobial susceptibility of *L. monocytogenes* isolates identified was carried out according to Clinical and Laboratory Standards Institute guidelines [25] via using the disk-diffusion on Mueller–Hinton agar (Oxoid CM0337) for different antimicrobial discs (Oxoid, Ltd.) of seven different classes of antibiotics. The antibiotics included Penicillin (P; 10 μg), Amoxicillin-Clavulanic acid (AMC; 20/10 μg), Cefotaxime (CTX; 30 μg), (Ceftazidime (CTZ; 30 μg), Amoxicillin (AX; 30 μg), Ciprofloxacin (CIP; 5 μg), Nalidixic acid (NA; 30 μg), Streptomycin (SM; 10 μg), Gentamicin (CN; 15 μg), Erythromycin (E; 10 μg), tetracycline (TET; 30 μg), Oxytetracycline (T; 30 μg), Vancomycin (VA; 30 μg), Chloramphenicol (C; 30 μg). *L. monocytogenes* isolates were evaluated as resistant, intermediate, or susceptible according to CLSI [25]. The categorization of the *L. monocytogenes* isolates as being multidrug resistant (MDR), extensively drug resistant (XDR), and pan-drug resistant (PDR) had been detected. Where the MDR microorganisms are resistant to at least one agent in three or more antimicrobial categories while XDR microorganisms are resistant to at least one agent in at least all but two or more antimicrobial categories and PDR microorganisms are resistant to all or nearly all available antimicrobials used.

### Multiple antibiotic resistance (MAR) index [26]

Multiple antibiotic resistance (MAR) index was calculated for all resistant *L. monocytogenes* isolates by dividing the number of drugs against which each strain displayed resistance above the total drugs tested (MAR Index = a/b), where “*a*” indicates the sum of test antibiotics the isolates displayed resistance to; “*b*” represents the total sum of antimicrobial agents used.

### Biofilm formation assay in vitro

Christensen’s test tube method was used to detect the qualitative assessment of the biofilm formation of *L. monocytogenes* in the current study [27]. Each *L. monocytogenes* strain was cultured in Brain Heart Infusion Broth (Oxoid Ltd) and uninoculated broth was used as a negative control. The tubes were incubated at 30 °C overnight. After incubation, each tube was emptied from the broth stained with 1% crystal violet, and incubated for 30 min. Finally, each tube was washed gently three times with sterile distilled water to remove non-adherent dye. Biofilm of *L. monocytogenes* that formed on the wall and bottom of the tube were stained purple. Biofilm formation assays were carried out two times.

### Quantitative risk assessment of poultry meat by *Listeria monocytogenes*

#### Hazard characterization

The hazard characterization of *L. monocytogenes* can be calculated using the Beta-Poisson dose-response (DR) models of Pouillot et al. [28] or of Xie et al. [29] using the following equation:

$${P}_I(d)=1-{\left(1+\frac{d}{\beta}\right)}^{-\alpha }$$

Where P1: is the probability of severe illness, d is the prevalence of the *Listeria monocytogenes* recovered in the samples examined in the current study, and α, β: infectious factors (constant, depending on pathogen) which were 0.52 and 0.43 according to Pouillot et al. [28] and 0.49 and 0.48 according to Xie et al. [29].

#### Exposure assessment

*Listeria monocytogenes* exposure assessment in poultry was calculated by the following eq. [30, 31]:

$$\textrm{Exposure}=\textrm{P}\times \textrm{C}\times \textrm{F}$$

Where P: represents the prevalence of the contamination of poultry samples by *L. monocytogenes* in the current study; C: the amount of poultry consumed per day per person in Egypt (https://www.fao.org/faostat/en/#country/59) and F: is the frequency of poultry consumption per year which is ranged from 20 to 30 times per year (http://www.fao.org/faostat/en/#data/QC/visualize).

#### Risk assessment [32]

The risk related to the consumption of food contaminated *by L. monocytogenes.* Was calculated using the following equation:

$$\textrm{Risk}=\textrm{Exposure}\times \textrm{P}1$$

Where P1 represents the probability of severe illness.

#### Statistical analysis

Statistical analysis to determine the correlation between biofilm formation and the drug resistance characteristics of the obtained isolates was analyzed using nonparametric statistical spearman correlation test using GraphPad PRISM® 9.1.2. (Graph Pad Software Incorporated, San Diego, USA). *P*-value < 0.05 was considered statistically significant.

## Results and discussion

### Prevalence of *L. monocytogenes* in poultry samples examined

The prevalence of *L. monocytogenes* detected in the current study was 8% (24/300 samples examined). The screening recovered *L. monocytogenes from* poultry carcasses 9.6% (24/250) only and there was no positive detection of it in hand swabs taken from the workers in poultry shops scattered in the city. Similar prevalence as 10% (5/50) of poultry samples was recorded to be contaminated with *L. monocytogenes* in previous study in Egypt [33], as well as 6.16% (9 of the 79 examined samples) in Turkey [34] and 9.4% (15/150) in Jordan [35]. While there was no *L. monocytogenes* could be isolated from raw chicken meat in Egypt previously in the study conducted by Dahshan et al., [36].

However slightly higher contamination rates by *L. monocytogenes* had been isolated from poultry carcasses as 17.9% (35/195) in Brazil [37], 18% (36/200) in Iran [38], 24.5% (13/53) in Italy [39], 19.2% (19/99) in Gauteng, South Africa [40], 20% (42/210) from poultry examined in Malaysia [41] and 38% (38/100) in Greece [42].

On the contrary, extremely higher rates of contamination of poultry carcasses and poultry processing environments were recorded by prevalence of 62.5% in Malaysia [43], as well as in Brazil as 52.83% [44] and 94.6% [45] in two different studies. It was confirmed that raw poultry meat is an appropriate environment for the existence of *L. monocytogenes* that can be retained in food and transferred to human via consumption of contaminated food [46]. The variation of *L. monocytogenes* contamination in different studies related to the source from where the bacteria were isolated, the geographical distributions, and the hygienic measures used for the food preparation system.

### Screening of different virulence genes in *L. monocytogenes*

**Fig. 1:**
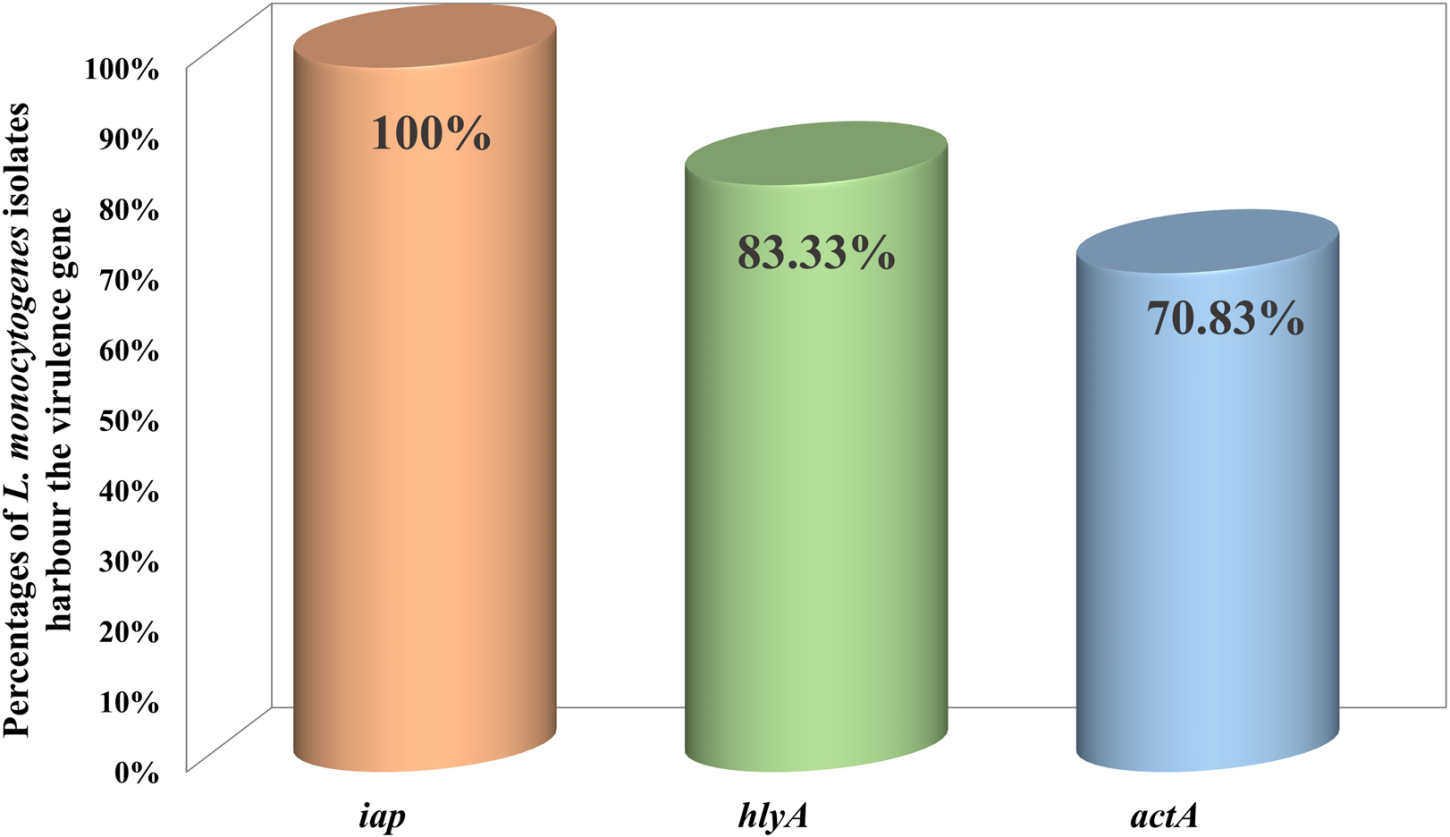
Percentages of *L. monocytogenes* isolates harbour the *iap, hylA and actA* virulence genes

Screening of three virulence genes *hylA*, *actA*, and *iap* which have key role in the pathogenesis of *L. monocytogenes,* for the confirmed *L. monocytogenes* isolated from the poultry carcasses, all the *L. monocytogenes* strains were positive for *iap gene,* while only 83.33% (20/24) were positive *hylA* gene and and *actA* gene was detected in 70.83% (17/24) of the isolates Fig. 1.

In Egypt, most detected virulence genes in *L. monocytogenes* strains are *hlyA, iap* and *actA* as recorded in 70.6, 70.6 and 52.9%, respectively, of the *L. monocytogenes* isolated from food samples previously [47], and as 100% of the samples harbored the three virulence genes in further study where the strains were isolated from meat, poultry meat, tilapia fish and raw milk [33]. In addition, the *hlyA and iap* virulence genes had been detected in all (100%) *L. monocytogenes* strains isolated from poultry meat (nine samples) in a previous study tookplace in Egypt [19]. However, in a previous study, that had taken place in Egypt, the *L. monocytogenes* isolated from fish samples were negative for all the mentioned genes [18]. *hylA* harbored *L. monocytogenes* strains were isolated from human samples before in Egypt [19], suggested a significant that may human infection resulted from the consumption of contaminated food which is a great threat that should not be neglected.

All *L. monocytogenes* strains isolated from 335 food samples including poultry meat in India harbored *actA, hlyA* and *iap* virulence genes [48]. Furthermore, it was reported that *hlyA* genes can be detected in *L. monocytogenes* recovered from food samples [49]. The high percentages of virulence genes in the bacterial strains isolated in the current study giving the bacteria capability to adhere, invade the epithelium of the human digestive system causing damage.

### Antibiogram of *L. monocytogenes* isolates from poultry samples examined

The antibiotic susceptibility of the twenty-four isolated *L. monocytogenes* strains, in the current study, in Table 2, showed high resistance to Tetracycline, β-lactams, and fluroquinolones classes of antibiotics where 91.67 and 83.33% of the isolates were resistant to tetracycline and oxytetracycline. In addition, approximately more than 50% of the strains showed resistance against β-lactams antibiotics including penicillin, amoxicillin, amoxicillin-Clavulanic acid, ceftazidime, and cefotaxime by prevalence 70.83, 70.83, 58.33, 58.33 and 45.83%, respectively. Half of the strains (50%) isolated were resistant to both ciprofloxacin and nalidixic acid. Lower antibiotics resistances were recorded for the remaining classes of antibiotics with the lowest resistance prevalence to chloramphenicol where 4 strains (16.67%) were resistant to it.

**Table 2:** Activities of antimicrobial agents tested against of the *listeria monocytogenes* isolates (*n* = 24) recovered from the poultry carcasses examined

Antibiotic name	μg/ disc	Antibiotic class	resistant	intermediated	susceptible
**Penicillin (P)**	10 μg	β-lactams	17 (70.83%)	0 (0%)	7 (29.16%)
**Amoxicillin-Clavulanic acid (AMC)**	20/10 μg	β-lactams	14 (58.33%)	3 (12.5%)	7 (29.16%)
**Cefotaxime (CTX)**	30 μg	β-lactams	11 (45.83%)	3 (12.5%)	10 (41.67%)
**Ceftazidime (CTZ)**	30 μg	β-lactams	14 (58.33%)	5 (20.83%)	5 (20.83%)
**Amoxicillin (AX)**	30 μg	β-lactams	17 (70.83%)	2 (8.33%)	5 (20.83%)
**Ciprofloxacin (CIP)**	5 μg	Fluoroquinolones	12 (50%)	3 (12.5%)	9 (79.17%)
**Nalidixic acid (NA)**	30 μg	Fluoroquinolones	12 (50%)	5 (20.83%)	7 (29.17%)
**Streptomycin (SM)**	10 μg	Aminoglycosides	8 (33.33%)	0 (0%)	16 (66.67%)
**Gentamicin (GEN)**	10 μg	Aminoglycosides	7 (29.16%)	1 (4.17%)	16 (66.67%)
**Erythromycin (E)**	15 μg	Macrolides	5 (20.83%)	0 (0%)	19 (79.17%)
**Tetracycline (TET)**	30 μg	Tetracycline	22 (91.67%)	0 (0%)	2 (8.33%)
**Oxytetracycline (T)**	30 μg	Tetracycline	20 (83.33%)	3 (12.5%)	4 (16.67%)
**Vancomycin (VA)**	30 μg	Glycopeptides	7 (29.17%)	3 (12.5%)	14 (58.33%)
**Chloramphenicol (C)**	30 μg	Chloramphenicol	4 (16.67%)	6 (25%)	14 (58.33%)

In Egypt, streptomycin, tetracycline, and β-lactams antibiotics are widely used not only for disease treatment but also for growth promotion and as prophylactic measures in the poultry industry sector with no regulation [50]. The irrational usage of antimicrobials leads to MDR acquisition in many pathogenic food poisoning bacteria such as *E. coli, Salmonellae* species, and *L. monocytogenes* as well as recovered from different sources of food samples including poultry, meat, and their products [19, 51, 52].

Likewise, our results, *L. monocytogenes* strains isolated from meat and environmental samples previously in Egypt, were MDR especially to penicillin, ampicillin, and tetracycline [19]. High resistance of *L. monocytogenes* strains was observed against oxytetracycline (76.4%), chloramphenicol (70.5%) with high susceptibility to erythromycin (64.6%), gentamicin (58.7%), and vancomycin (58.7%) where bacteria isolated from food products from Egypt, previously [47] which were corresponded to the current study results. Similarly, all *L. monocytogenes* strains isolated from chicken in Makurdi Metropolis, Nigeria were resistant to amoxicillin, cloxacillin, and tetracycline [53].

Contrary to our results, all *L. monocytogenes* strains isolated from chicken in northern Greece were sensitive to ampicillin, cephalothin, amoxicillin, ciprofloxacin, penicillin, cefotaxime, chloramphenicol, gentamicin, enrofloxacin, erythromycin, kanamycin, neomycin, vancomycin, streptomycin, and sulfamethoxazole-trimethoprim [42] while all the strains were resistant to nalidixic acid. Furthermore, all *L. monocytogenes* strains isolated from chicken in Makurdi Metropolis, Nigeria were susceptible to gentamycin, erythromycin, and chloramphenicol [53]. In addition to the results of the previous study where 100% of *L. monocytogenes* strains isolated from poultry slaughtered and sold in Brazil were sensitive to tested antibiotics, except for clindamycin, where 5% of the isolates were resistant [37]. The antibiotic profile of *L. monocytogenes* strains variation from one study to another is related to the different samples from which the bacteria were isolated, the country, and the usage regulation correlated to each country.

### Antimicrobial resistance profiles of *L. monocytogenes*

The Multiple Antibiotic Resistance (MAR) index of *L. monocytogenes* strains tested in the current study was ranged from 0.14 to 0.86 with an average 0.47 (Table 3). The majority (95.83%, 23/24) of *L. monocytogenes* strains showed resistance to three or more tested antibiotics, in which the MAR index value was higher than 0.2, indicating the overuse of antibiotics. However, only 4.2% (1/24) of the stains had MAR value of 0.14. Consistent results had been recorded previously where 70.5% of the *L. monocytogenes* strains isolated from vegetable farms in Malaysia had MAR index ranged from 0.22 to 0.56 and 29.5% of the strains had MAR lower than 0.2 [54]. However, all (100%) *L. monocytogenes* strains isolated from raw meat in Northwestern Nigeria had MAR value ranged from 0.27 to 0.73 [55]. On the other hand, only 29.2% of the *L. monocytogenes* strains isolated from raw burger patties in Malaysia had MAR more than 0.2 value with 39% had MAR index lower than 0.2 with 31.7% has no resistance to any antibiotic tested [56]. Difference in MAR values among the different studies related to many factors such as the antibiotics used, the source of samples, the geographical changes and the most important reason linked to the antibiotics used for the animal, human and environment and it was published that resistant bacteria had MAR index higher than 0.2 originated from the overuse of antibiotic drugs for all the source [26].

**Table 3:** Antimicrobial resistance patterns and Multiple Antibiotic Resistance (MAR) index for the *Listeria monocytogenes* isolates (*n* = 24)

Antibiotics Resistance Pattern	No. of strains	MAR index	Classification of the strains	
			Type of resistance	No. of isolates (%)
TET, T, P, AX, AMC, CTZ, CIP, NA, CTX, SM, GEN, VA	4	0.86	Extensively drug-resistant	4 (16.7%)
TET, T, P, AX, AMC, CTZ, CIP, NA, CTX, VA	1	0.71	Multi-drug resistant	19 (79.2%)
TET, T, P, AX, AMC, CTZ, CIP, NA, C, E	2	0.71		
TET, T, P, AX, AMC, CTZ, CIP, NA, CTX	3	0.64		
TET, P, CTX, CTZ, SM, GEN, E, C	1	0.57		
TET, T, P, AX, AMC, CTZ, C, E	1	0.57		
P, CTX, SM, GEN, VA	2	0.36		
TET, T, P, AX, CTZ	2	0.36		
TET, T, P, AX, CIP	1	0.36		
TET, T, AX, AMC	3	0.29		
TET, T, CIP, E	1	0.29		
TET, T, NA	2	0.21		
TET, SM	1	0.14	Low-drug resistant	1 (4.2%)

### Categorization of *L. monocytogenes* isolates based on their antimicrobial resistance profiles

*L. monocytogenes* strains (*n* = 24) isolated from poultry meat in the present study were categorized tested as pan-drug-resistant (PDR), Extensively drug-resistant (XDR), Multidrug resistant (MDR) and low-drug resistant (LDR), depending to their resistance against the 14 different antibiotics according to the description of Magiorakos et al. [57] in Table 3. Nineteen strains of *L. monocytogenes* (79.2%) were classified as Multidrug-resistant (MDR) as they exhibited resistance to three or more classes of antibiotics None of the tested isolates were resistant to all antibiotics tested. Extensively drug-resistant (XDR) *L. monocytogenes* strains represented 16.7% (4/24) and one strain was classified as low-drug resistant (LDR) with no strain under named as pan-drug-resistant (PDR). Many studies isolated *L. monocytogenes* of multidrug resistant type by high prevalence as 100% *L. monocytogenes* isolated from raw meat in Nigeria [55] and from chicken meat in Malaysia [58]. On the contrary, much lower prevalence of MDR among *L. monocytogenes* strain recovered from raw meat and retail foods such as 18.9% [59] and 20% [37]. *L. monocytogenes* is considered one of the food-borne pathogens associated with many outbreaks all over the world [10] due to the spread of MDR and XDR strains leaving few options for treatments.

### Biofilm formation capability of *L. monocytogenes*

**Fig. 2:**
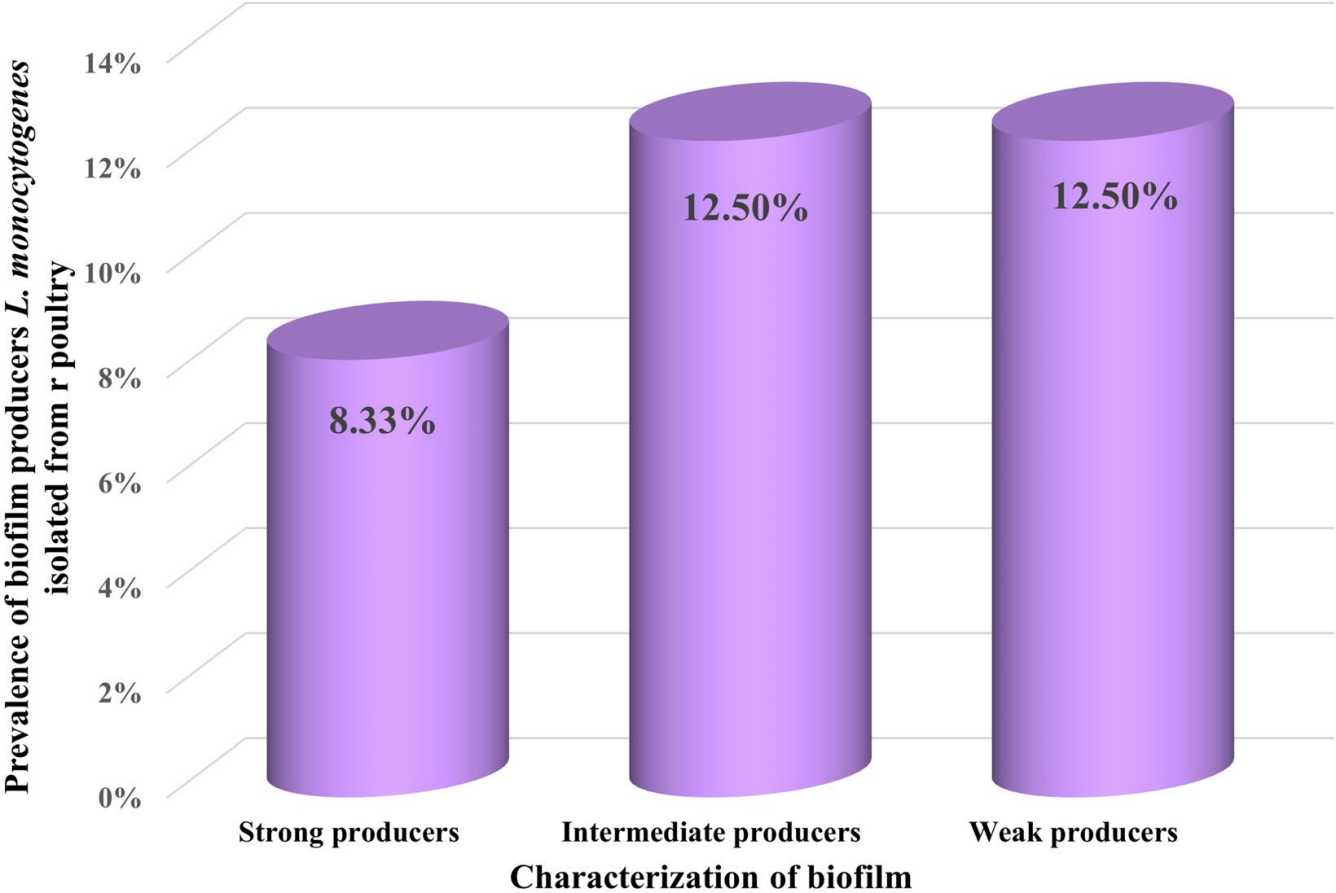
Prevalence of weak, intermediate and strong biofilm producers *L. monocytogenes* isolates

The biofilm capability of *L. monocytogenes* isolates recovered in the current study from poultry samples was categorized as strong producers in 8.33%, intermediated producers in 12.5% and weak producers in 12.5% of the recovered samples (Fig. 2). It was obvious that the majority percentages of *L. monocytogenes* produce weak to moderate biofilm producers. There was a great significant (< 0.0001) correlation between the biofilm formation and the antibiotic resistance characteristics of the isolates. Previous studies illustrated that bacterial cells of *L. monocytogenes* that were isolated from different sources including clinical, meat, and milk samples were generally weak to moderate biofilm producers [60–62]. In Egypt, in a previous study, the biofilm formation of *L. monocytogenes* isolated from different sources including humans, animals, food, and environment was investigated as moderate to strong [10]. Biofilm formation by foodborne pathogens such as *L. monocytogenes* represents a serious concern in the food industry [63]. However, there are scarcity of data about the biofilm capability of *L. monocytogenes* isolated in Egypt. Therefore, more research that focuses on the characterization of the biofilm formation and persistence of such pathogens is needed.

### Risk assessment of consumption of poultry contaminated by *L. monocytogenes*

**Fig. 3:**
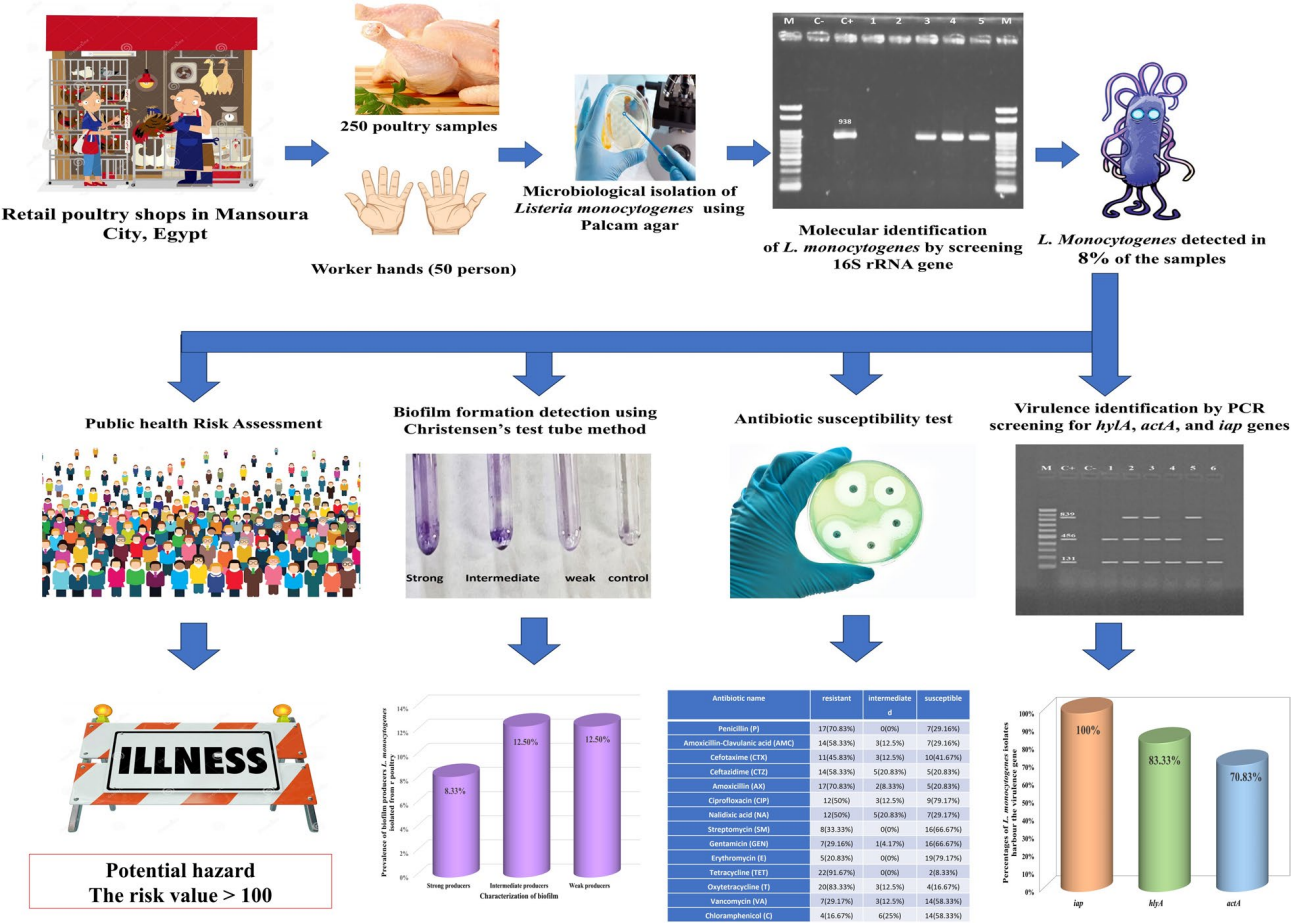
The whole work flow of the current study including the results highlighting the potential human risk illness associated to consumption of contaminated poultry with *L. monocytogenes* isolates

The calculated probability of illness associated with the consumption of the current study poultry samples contaminated with *L. monocytogenes* was 0.9 to 0.92 which is considered too high especially when the exposure assessment of *L. monocytogenes* ranged from 112.03 to 168.04 g per year. Therefore, the risk associated with the consumption of *L. monocytogenes* had a value exceeding 100 which reflects the potential hazard of such food origin and represents a significant level of risk of *L. monocytogenes* highlighting the importance of preventive measures to minimize exposure and protect public health. The current study as shown in Fig. 3 is the first study in Egypt to measure the risk assessment associated with the consumption of poultry samples contaminated with *L. monocytogenes* illustrating the antimicrobial susceptibility profile, virulence and biofilm formation capability of the isolates highlighting the importance of continuous monitoring of such pathogens in poultry industry*.* Therefore, future research quantifies the risk assessment through the whole poultry processing chain from farm to fork in Egypt is needed.

## Conclusion

The current study demonstrated that poultry can be a vector for *L. monocytogenes* as a major contaminant to human consumers, even handlers, and the surrounding environment. Such results require great attention to the awareness of hygienic measures in the food industry. The majority of *L. monocytogenes* isolated in the present study were multidrug-resistant, holding virulence factors including their biofilm formation capability, adding further burden to the existing global antimicrobial resistance problems besides the risk of human infection incidence of difficult or no treatment. The molecular characteristics of the *L. monocytogenes* strains isolated from poultry had the same molecular features of clinical samples isolated strains in Egypt, indicating that poultry could be a critical source of human infections since they harbor multi-virulent multi-drug resistant *L. monocytogenes* strains. Therefore, the establishment of control systems to monitor the use of antibiotics in veterinary medicine is crucial and should be regularly monitored.

## Data Availability

All data and materials used during this study are included in this published article.
